# Supplementary material for: Microbial metabolism: optimal control of uptake versus synthesis
Source: PeerJ. 2014 Feb 25;2:e267. doi: 10.7717/peerj.267 (PMC3940620; doi:10.7717/peerj.267)
Supplement: Supplemental Information 1 [file peerj-02-267-s001.pdf]

SUPPLEMENTAL INFORMATION

Microbial metabolism: optimal control of uptake versus synthesis

Steven A. Frank*

November 4, 2013

- The first section presents the supplemental figures mentioned in the text.
- The second section presents methods for analyzing optimality when genetic mixture occurs within patches.
- The third section emphasizes that clones inevitability produce mutants within local populations, causing genetic mixture. Thus, optimality analyses must account for competition and potential cooperation between different genotypes. The consequences of genetic variability typically depend on demography.
- The fourth section considers how to analyze cases in which microbes can adjust their traits, such as uptake versus synthesis, in response to changing conditions.

---

*Department of Ecology and Evolutionary Biology, University of California, Irvine, CA 92697–2525, USA, email: safrank@uci.edu

1 Supplemental figures

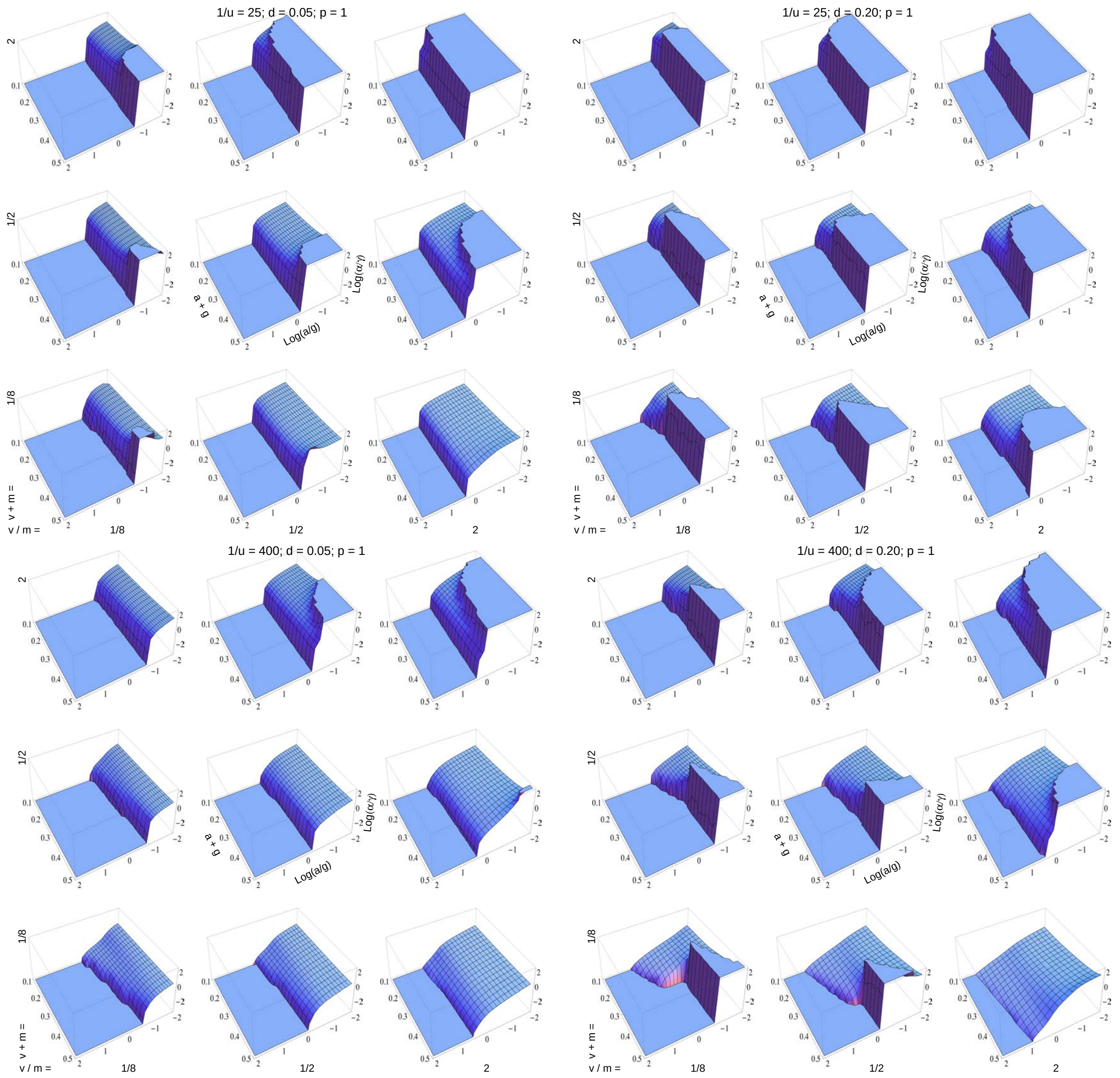

Supplemental Figure 1. Optimal control variables for uptake, α, and synthesis, γ. Same as Fig. 2, with varying values of average patch lifespan, $1/u$, and cell death rate, d. Plots discussed in main text.

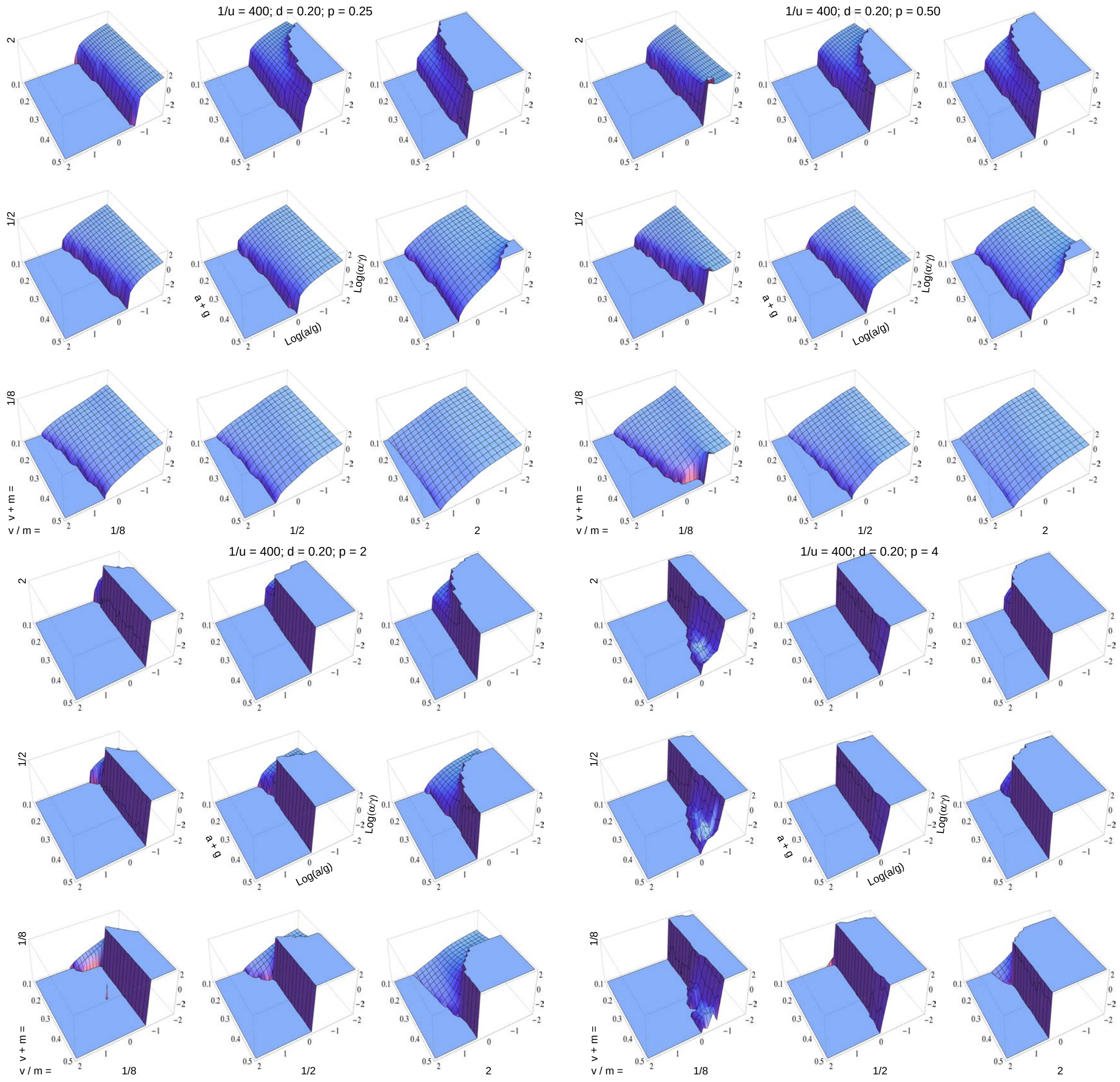

Supplemental Figure 2. Optimal control variables for uptake, α, and synthesis, γ. Same as lower-right array in Supplemental Fig. 1, with varying values of p, the rate of decay of the metabolic factor within cells. Plots discussed in main text.

2 Mixed genetic structure

The Discussion in the main text suggested that genetic mixture can powerfully affect how natural selection shapes trait values. Put another way, the objective function in optimality studies must account for genetic mixture. Here, I add a few technical comments.

An approximate objective function can be formed by starting with an expression for fitness, w, that measures the success of a particular genotype. Let x be the traits to be optimized, in which x may be a vector of multiple trait values that must be optimized simultaneously. Following the Discussion in the text, with additional detail here, we focus on a common genotype and an alternative rare genotype. Let the traits of the common genotype be x^*, and the traits of the rare genotype be $\hat{x}$. When genetic mixtures occur, the common genotype is almost always with another common genotype. We write the fitness of that common genotype paired with itself as $w(x^*, x^*)$. In other words, we calculate the aggregate fitness of the common type by analyzing its success as a clone.

By contrast, the rare type will occur in two different kinds of patches. With probability r, the rare type will settle in a patch with another rare type, leading to a fitness of $w(\hat{x}, \hat{x})$. With probability $1 - r$, the rare type pairs with the common type, leading to a fitness for the rare type in that pairing of $w(\hat{x}, x^*)$. The aggregate fitness of the rare type is the average of the two patch compositions, $rw(\hat{x}, \hat{x}) + (1 - r)w(\hat{x}, x^*)$. Here, r is the spatial correlation between genotypes within patches, which is equivalent to the genetic coefficient of relatedness used in studies of kin selection and social evolution (Hamilton, 1970; Frank, 1998). More mixing between genotypes reduces r.

How can we find an optimum value for x? A possible optimal type would be one for which fitness when common is greater than any rare alternative (**maynard-smith82evolution**). Here, optimality simply means evolutionary stable when common against any rare genetic variant. Using that criterion, we must find x^* such that

$$w(x^*, x^*) > rw(\hat{x}, \hat{x}) + (1 - r)w(\hat{x}, x^*)$$

for any rare type with traits $\hat{x} \neq x^*$ (Frank, 1998, 2010b). It is often sufficient to compare a candidate for the optimum, x^*, to values that deviate by a small amount from that candidate, $\hat{x} = x^* \pm \epsilon$ for small ϵ. In that case, searching for x^* is relatively easy when x represents a single trait value, leading to a one-dimensional optimization problem. For a candidate x^*, one simply checks fitness against values of $\hat{x} \pm \epsilon$.

For multidimensional problems, one must consider trait deviations from a candidate x^*, against all possible multidimensional deviations. That comparison may be difficult, because even for small deviations, there are infinite combinations of trait deviations. On can simplify a bit by searching on a sphere centered at x^* and having radius ϵ. But even that simplified search may sometimes be complicated. Various multidimensional heuristic search methods may be tried. Explicit dynamical models, numerical methds or stochastic simulations of populations may be necessary to evaluate the range of assumptions over which the heuristic search approaches provide a good approximation of optimal trait values.

3 Long-lived clones: competition from local mutants

Large microbial population size means that mutants inevitably arise within each local population. Those mutants create genetic variability and local competition between different genotypes. If local populations have sufficiently long lifespans, an initial clone certainly faces competition from its own descendant mutants. That internal competition may become a dominant force shaping the evolution of regulatory controls over metabolism (Frank, 2010a, 2010b, 2013; Diard et al., 2013). Thus, the evolutionary design of metabolism often depends strongly on interactions between demography and genetic variability.

4 Feedback control and conditional expression

My analysis assumed that the control variables for uptake and synthesis evolved to fixed levels of expression in each cell in response to constant environmental conditions. Alternatively, the explicit dynamics of uptake and synthesis may be regulated in response to changing conditions, allowing cells to adjust expression levels to varying conditions.

In the dynamics of eqn 1 in the main text, the uptake and control variables, α and γ, evolve to constant levels of expression. Alternatively, we may consider control variables that change dynamically, for example by

$$\dot{\alpha} = r_0 + r_1 B - r_2 \gamma \alpha - r_3 I \alpha - r_4 \alpha$$

$$\dot{\gamma} = s_0 + s_1/(1+I) - s_2 \alpha \gamma - s_3 B \gamma - s_4 \gamma.$$

This expression changes the evolutionary problem by treating the original variables α and γ as dynamically controlled consequences of the new sets of control variables, $\{r_i\}$ and $\{s_i\}$. The levels of uptake and synthesis, α and γ, now respond dynamically to changing conditions in a manner controlled by $\{r_i\}$ and $\{s_i\}$. A full analysis would consider the costs associated with each new control variable, and how those costs influence the tendency to respond to changing conditions.

One may obtain the constant control variables of the main text as a special case by setting $r_1 = r_2 = r_3 = 0$, so that uptake approaches the constant equilibrium value $\alpha = r_0/r_4$, with a similar assumption leading to $\gamma = s_0/s_4$. Under those assumptions for nearly constant control variables, simultaneous optimization of (r_0, r_4) and (s_0, s_4) should give the same results as in the main text. I tested a few cases and did obtain a match.

References

Diard, M., Garcia, V., Maier, L., Remus-Emsermann, M. N. P., Regoes, R. R., Ackermann, M. & Hardt, W.-D. 2013. Stabilization of cooperative virulence by the expression of an avirulent phenotype. *Nature* 494:353–356.

Frank, S. A. 1998. *Foundations of Social Evolution.* Princeton, New Jersey: Princeton University Press.

Frank, S. A. 2010a. Microbial secretor–cheater dynamics. *Philosophical Transactions of the Royal Society B* 365:2515–2522.

Frank, S. A. 2010b. The trade-off between rate and yield in the design of microbial metabolism. *Journal of Evolutionary Biology* 23:609–613.

Frank, S. A. 2013. Microbial evolution: regulatory design prevents cancer-like overgrowths. *Current Biology* 23:R343–R346.

Hamilton, W. D. 1970. Selfish and spiteful behaviour in an evolutionary model. *Nature* 228:1218–1220.
